# A study of hospitalized COVID-19 patients with AKI in a setting of multiracial developing country

**DOI:** 10.1186/s12882-024-03498-x

**Published:** 2024-04-05

**Authors:** S. H. Ooi, K. P. Ng, Pavai Sthaneshwar, S. K. Lim, P. Y. Khor, J. Y. Lim, W. S. Siow, K. W. Lim, Muhummad Azlan

**Affiliations:** 1 Internal Medicine Department, University Malaya Medical Center, Kuala Lumpur, Malaysia; 2 Pathology Department, University of Malaya, Kuala Lumpur, Malaysia

**Keywords:** Covid-19, Acute kidney injury, Multiracial developing country

## Abstract

**Background:**

The commonest indication for hospitalization in COVID-19 patients is hypoxemia or severe respiratory symptoms. However, COVID-19 disease may result in extrapulmonary complications including kidney-related pathology. The reported incidence of renal involvement related to COVID infection varies based on geographical location.

**Objective:**

This study aimed to assess the incidence rate of AKI in hospitalized COVID-19 patients and identify risk factors and prognostic predictors.

**Method:**

In this retrospective study, we recruited hospitalized COVID-19 patients from January 2021 until June 2021 at the University Malaya Medical Center. The inclusion criteria were hospitalized for ≥ 48 h with confirmed COVID-19 infection and at least 18 years old. Patient demographic and clinical data were collected from electronic medical records. The staging of AKI was based on criteria as per KDIGO guidelines.

**Results:**

One thousand five hundred twenty-nine COVID patients fulfilled the inclusion criteria with a male-to-female ratio of 759 (49.6%) to 770 (50.3%). The median age was 55 (IQR: 36–66). 500 patients (32.7%) had diabetes, 621 (40.6%) had hypertension, and 5.6% (*n* = 85) had pre-existing chronic kidney disease (CKD). The incidence rate of AKI was 21.1% (*n* = 323). The percentage of COVID patients in different AKI stages of 1,2 and 3 were 16.3%, 2.1%, and 2.7%, respectively. Fifteen hospitalized patients (0.98%) required renal replacement therapy. 58.8% (*n* = 190) of AKI group had complete recovery of kidney function.

Demographic factors included age (*p* < 0.001), diabetes (*p* < 0.001), hypertension (*p* < 0.012), CKD (*p* < 0.001), and vaccination status (*p* = 0.042) were associated with an increased risk of developing AKI. We found that the AKI cohort had statistically significant lower platelet counts and higher ferritin levels than the non-AKI cohort. AKI is a risk predictor of prolonged hospitalization (*p* < 0.001) and higher mortality rates (*P* < 0.001).

**Conclusion:**

AKI is a common clinical complication among hospitalized COVID-19 patients. The etiology of AKI is multifactorial and may have an adverse impact on patient morbidity and mortality.

## Introduction

The emergence of the COVID-19 pandemic infection since 2019 has resulted in millions of worldwide morbidity and mortality [1, 2]. As this is a novel and acute onset of a global catastrophic event with minimal understanding of the disease pathophysiology at an early stage, medical professionals, including nephrologists, are tasked to study the clinical spectrum of COVID-19 with the hope of curtailing the transmission of the virus and improving the care of patients.

COVID-19 infection has different clinical phenotypes and has been described as a multisystem inflammatory syndrome, thus posing a great challenge to primary physicians. The presentation can be asymptomatic, symptomatic only needing outpatient treatment or symptomatic necessitating hospitalization. This disease predominantly affects the pulmonary system and causes respiratory distress, being the main cause of hospital admission. Other organ involvements have been well reported, including renal, hematology, gastroenterology, ophthalmology, neurology, and cardiovascular system [3–7]; however, the described extrapulmonary system manifestation was only readily detected in hospitalized patients, particularly renal complication that can often be subclinical and encompass different mechanisms and histo-pathological changes such as acute tubular necrosis (ATN), immune- mediated inflammation (e,g glomerulonephritis) and direct viral, toxic effects on the kidney.

Malaysia is a multiracial country with diverse ethnic groups and socioeconomic backgrounds making it an ideal environment to evaluate the disease across a range of demographic in particular, the ethnicity factor. In our country, the first confirmed COVID-19 infection was on 25^th^ January 2020 and sparked a spike since March 2020 that continues to spiral upwards [8]. Kuala Lumpur is the capital of Malaysia, with a dense population of 2.0 million people [9]. University Malaya is an academic tertiary hospital in Kuala Lumpur and has been actively managing COVID-19 patients since the beginning of February 2020. The data on the incidence of AKI in confirmed COVID-19 infection is scarce in Malaysia. Hence, this study aims to look at the incidence rate of AKI in hospitalized COVID-19 patients in a multiracial country and identify the risk predictors.

## Method

**Fig. 1:**
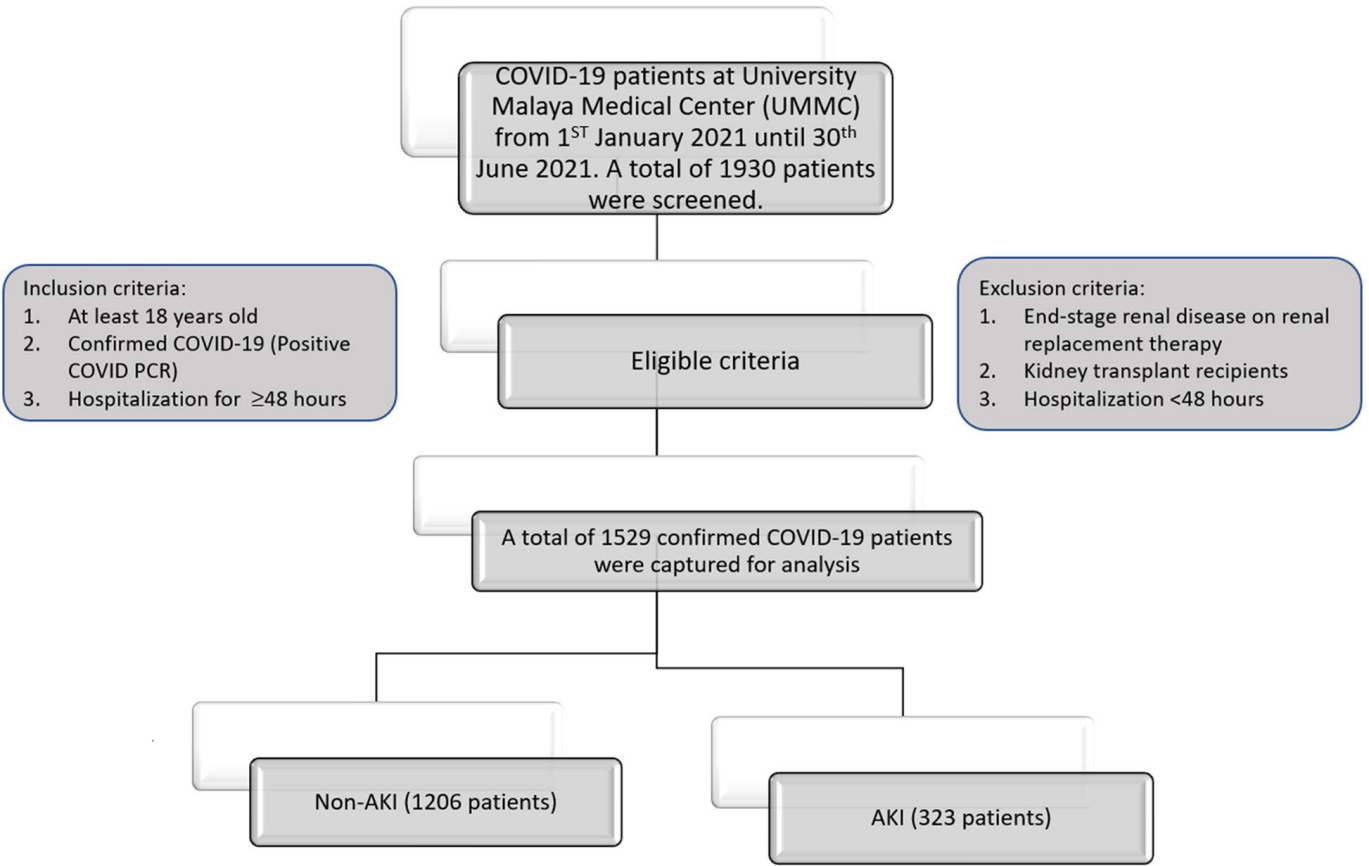
Workflow diagram of the patient selection

This is a single-center retrospective cohort study of hospitalized COVID-19 patients at the University Malaya Medical Center. The inclusion criteria are patients at least 18 years old and hospitalized for at least 48 hours for confirmed COVID-19 from 1 January 2021 until 30 June 2021. A confirmed COVID-19 is defined as those with a positive real-time COVID polymerase chain reaction (PCR) test. The exclusion criteria are individuals with an end-stage renal disease (ESRD) on maintenance hemodialysis, kidney transplant recipients, those with fewer than two samples of renal profile throughout hospitalization, and those hospitalized for less than forty-eight hours. The workflow diagram for this study is illustrated in Fig. 1. Patients' basic demographic, clinical, biochemical, and radiological parameters were collected from electronic medical records (EMR). The patient's progress was tracked until the day they were discharged from the hospital.

The baseline creatinine is defined as the serum creatinine level obtained three months prior to admission. The on-admission serum creatinine served as the baseline for patients without previous serum creatinine measurements. The highest serum creatinine recorded throughout hospitalization for COVID-19 is noted as the peak serum creatinine. Patients who undergo repeated serum creatinine testing at least two weeks up to three months after discharge at the outpatient clinic are defined as post-discharge serum creatinine level.

The definition of acute kidney injury was based on the KDIGO-AKI guidelines; stage 1 is the serum creatinine of at least 26 umol/l or 1.5–1.9 times from baseline, stage 2 is 2.0–2.9 times from baseline serum creatinine, and stage 3 is ≥ 3 times from baseline serum creatinine or serum creatinine of > 354 umol/L or the need of renal replacement therapy. Our study defines AKI recovery as a patient who had AKI and whether the serum creatinine returned to baseline or normalized during the post-discharge creatinine level. Urine FEME is employed to assess the severity of proteinuria (1 + to 3 +), hematuria (erythrocytes ≥ five cells/ul), and leukocyturia (significant if leucocyte ≥ five cells/ul). The non-invasive ventilator usage refers to the utilization of bilevel positive airway pressure (BiPAP) or continuous positive airway pressure (CPAP).

### Statistical analysis

Data was analyzed using IBM SPSS Statistic 26 (SPSS, Inc., Chicago, IL, USA). Normal distributed continuous data will be recorded as mean ± two standard deviations (2SD), and categorical data will be recorded as absolute values with percentages. Non-normally distributed continuous data will be recorded as median with interquartile range (IQR). Continuous variables between groups will be compared using the Student's t-test or Mann–Whitney test, whereas the chi-square test is utilized for categorical variables. Pearson's and partial correlation analyses assessed the association between continuous variables. Multivariate logistic regression will be used to examine the prediction of AKI. The Receiver Operating Characteristics (ROC) curve plot was performed to determine the capacity of serum ferritin to predict AKI. When suitable, cut-of values were derived from the point on the ROC curve with minimum distance to the upper-left corner. The Kaplan–Meier method was used to plot and analyze survival curves and to provide survival rates reported as Kaplan–Meier estimates, including a 95% confidence interval in square brackets. A comparison of the survival curves was performed using the log-rank test.

## Result

A total of 1529 patients were captured in our study. The median age was 55 years old (interquartile range: 36–66), with a male-to-female ratio of 759 (49.6%) and 770 (50.4%). Malay ethnicity (*n* = 909) was predominant, with 59.5%, followed by Chinese, Indian, and others at 20.3%, 16%, and 4.3%, respectively. Out of 1529 patients, 500 had diabetes (32.7%), 621 had hypertension (40.6%), and 85 had chronic kidney disease (5.6%) for the comorbidities. 182 out of 1,529 patients (12%) had COVID vaccination prior to admission for confirmed COVID-19 infection. Our study's incidence rate of AKI was 21.1% (*n* = 323). The proportion of different AKI stages of 1,2 and 3 were 16.3%, 2.1%, and 2.7%, respectively. A total of 317 patients had urine analysis sent, 140 patients had proteinuria (44%), 148 patients had microscopic hematuria (46%), and 96 had leukocyturia (30%). Table 1: Patient demographic characteristics as stated.

**Table 1:** Basic demographic characteristics

Basic characteristic	Number (*n* = 1529), (%)
Gender	
Male	759 (49.6%)
Female	770 (50.4%)
Age	55 (IQR:36–66)
Race	
Malay	909 (59.5%)
Chinese	310 (20.3%)
Indian	244 (16%)
Others	66 (4.3%)
Comorbidities	
Diabetes	500 (32.7%)
Hypertension	621 (40.6%)
Chronic kidney disease	85 (5.6%)
Medications	
ARB/ACE inhibitor	278 (18.2%)
SGLT2	34 (2.2%)
Metformin	303 (19.8%)
NSAID	128 (8.4%)
Acute kidney injury	
No	1206 (78.9%)
Yes	323 (21.1%)
Stages AKI (KDIGO, 2012)	
1	250 (16.3%)
2	32 (2.1%)
3	41 (2.7%)
Dialysis	
No	1514 (99.02%)
Yes	15 (0.98%)
AKI recovery	
No	133 (41.2%)
Yes	190 (58.8%)
ICU admission	
No	1199 (78.4%)
Yes	330 (21.6%)
Covid vaccination	
No	1337 (88%)
Yes	182 (12%)
Types of vaccination	
Pfizer	127 (8.4%)
AstraZeneca	27 (1.8%)
Sinovac	28 (1.8%)
Number of vaccinations	
One dose	72 (4.7%)
Two doses	107 (7.1%)
Three doses	3 (0.2%)
Death	
No	1394 (91.2%)
Yes	135 (8.8%)
Pneumonia	
No	602 (39.4%)
Yes	924 (60.6%)
Ventilation	
No	1403 (91.8%)
Yes	126 (8.2%)
Types of ventilation	
Invasive ventilation	94 (6.2%)
Non-invasive ventilation	32 (2%)

AKI patients had higher baselines of creatinine (94 vs. 62; *p* < 0.001) and peak serum creatinine (107 vs 61; *p* < 0.001). Urinary abnormalities such as proteinuria, microscopic hematuria, and leukocyturia in 66.7%, 64.4%, and 37.6% were significantly greater in the COVID-19 with AKI patients. A total of 15 hospitalized patients (0.98%) needed dialysis. Recovery of AKI occurred in 190 patients (58.8%), whereas 133 (41.2%) patients had no renal recovery during the clinic follow-up.

The median hospitalization stay was fifteen days for patients with AKI and eight days for the non-AKI group; *P* < 0.001. We found male gender had a higher percentage (64.7%) than females (35.3%) in the AKI group; *P* < 0.001. The patient's median age was 66 in the AKI group, whereas it was 49 in the non-AKI group. Among 182 patients with COVID-19 vaccination, 13.3% (*n* = 159) didn't develop AKI throughout hospitalization. In those who had covid vaccination and non-AKI, 9.6% had Pfizer (*p* = 0.008), 1.8% had AstraZeneca (*p* = 0.988), and 1.8% had the Sinovac vaccine (*p* = 1.00). Lower median serum lymphocytes (1.13 × 10^9^) and platelet count (203 × 10^9^) with reciprocal higher serum ferritin (747 ug/L) were found to be higher in the AKI patients.

**Fig. 2:**
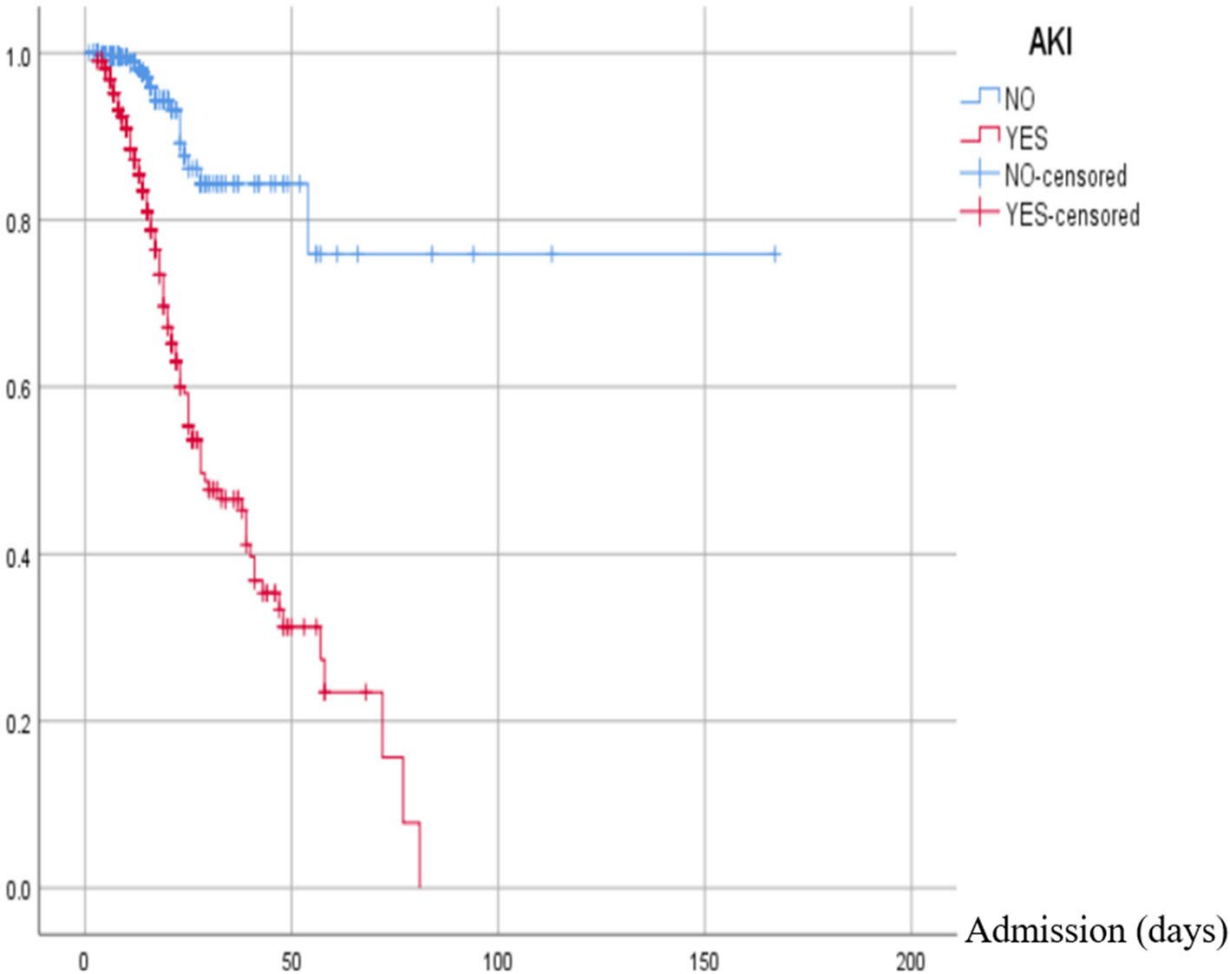
Kaplan–Meier survival curve for patients with and without AKI. The red line indicates patients with AKI, and the blue line is for those without AKI

One hundred fifty-one patients (46.7%) with AKI had ICU admission; *p* < 0.001. Among 924 patients who had pneumonia, of whom 288 (31.2%) patients had AKI and 636 (89.7%) patients had no AKI. 30.3% (*n* = 98) needed ventilation, and 25.7% (*n* = 83) needed vasopressor use in COVID-19 with AKI. 135 (8.8%) of the total hospitalized patients unfortunately passed away, and 83% (*n* = 112) of the deceased patients had AKI during hospitalization. Table 2 states the summary of the patient’s characteristics with and without AKI. Patient with AKI has significantly less survival compared to non-AKI patients (log-rank of 84.34; *p* < 0.001). AKI stages stratify the result of the Kaplan–Meier survival analysis are presented in Fig. 2.

**Table 2:**
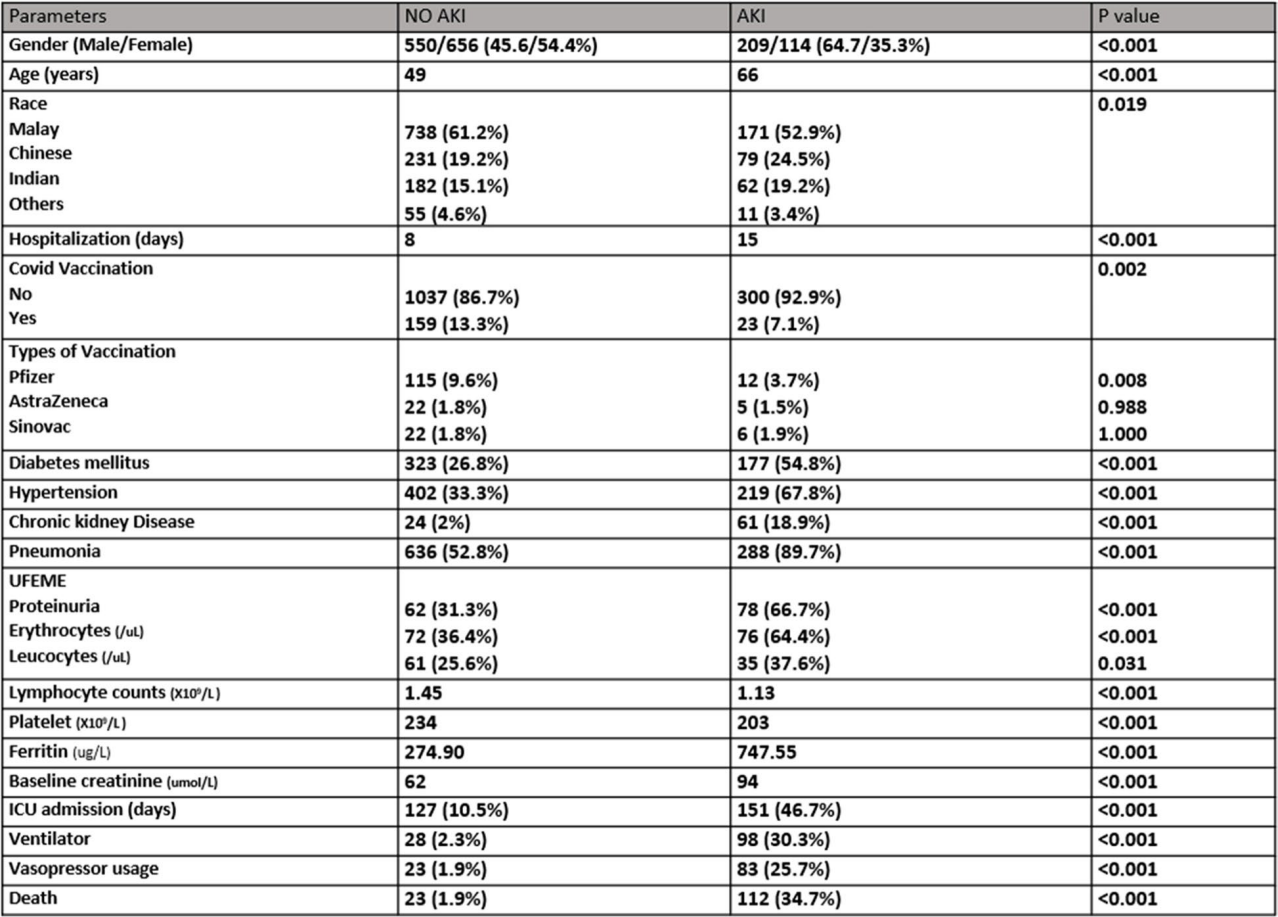
Patients characteristics without and with AKI

The predictors of AKI in our study were age odd ratio [OR] 1.04; 95% confidence interval [95% CI] 1.03–1.054, chronic kidney disease (OR 5.26; 95% CI 3.08–8.98), diabetes (OR 1.75; 95% CI 1.29-2.37), hypertension (OR 1.56; 95% CI 1.10-2.21), Covid-19 vaccination (OR 0.58; 95% CI 0.34-0.98), platelet (OR 0.99; 95% CI 0.99-1.00) and ferritin level (OR 1.00; 95% CI 1.00-1.00).

**Fig. 3:**
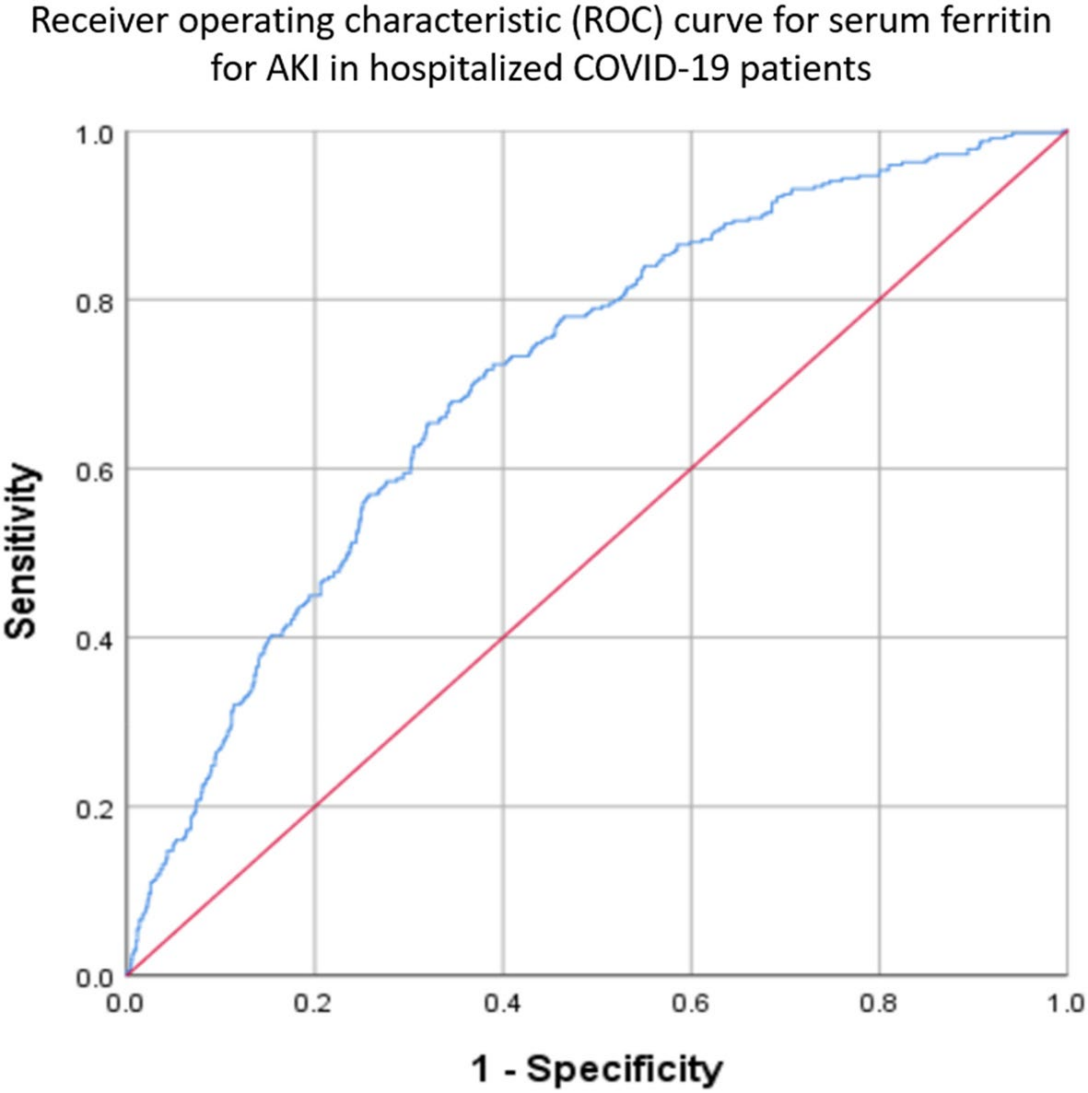
Serum ferritin ROC for AKI. The AUC for this ROC was 0.711 with the optimal cutoff of 500 ug/L with a sensitivity of 66% and specificity of 66%, PPV: 34%, NPV: 87% *p* < 0.001. *Positive predictive value (PPV), Negative predictive value (NPV)

The summary of the univariate and multivariate logistic regression for AKI in hospitalized COVID-19 patients is stated in Table 3. The area under the curve (AUC) for serum ferritin was 0.711, a cutoff of 500 ug/L with a sensitivity of 66% and specificity of 66%; positive predictive value (PPV) of 34%, and negative predictive value (NPV) of 87%; *p* < 0.001. The receiver operator curves (ROC) for serum ferritin in predicting the AKI among hospitalized COVID-19 patients are shown in Fig. 3.

**Table 3:**
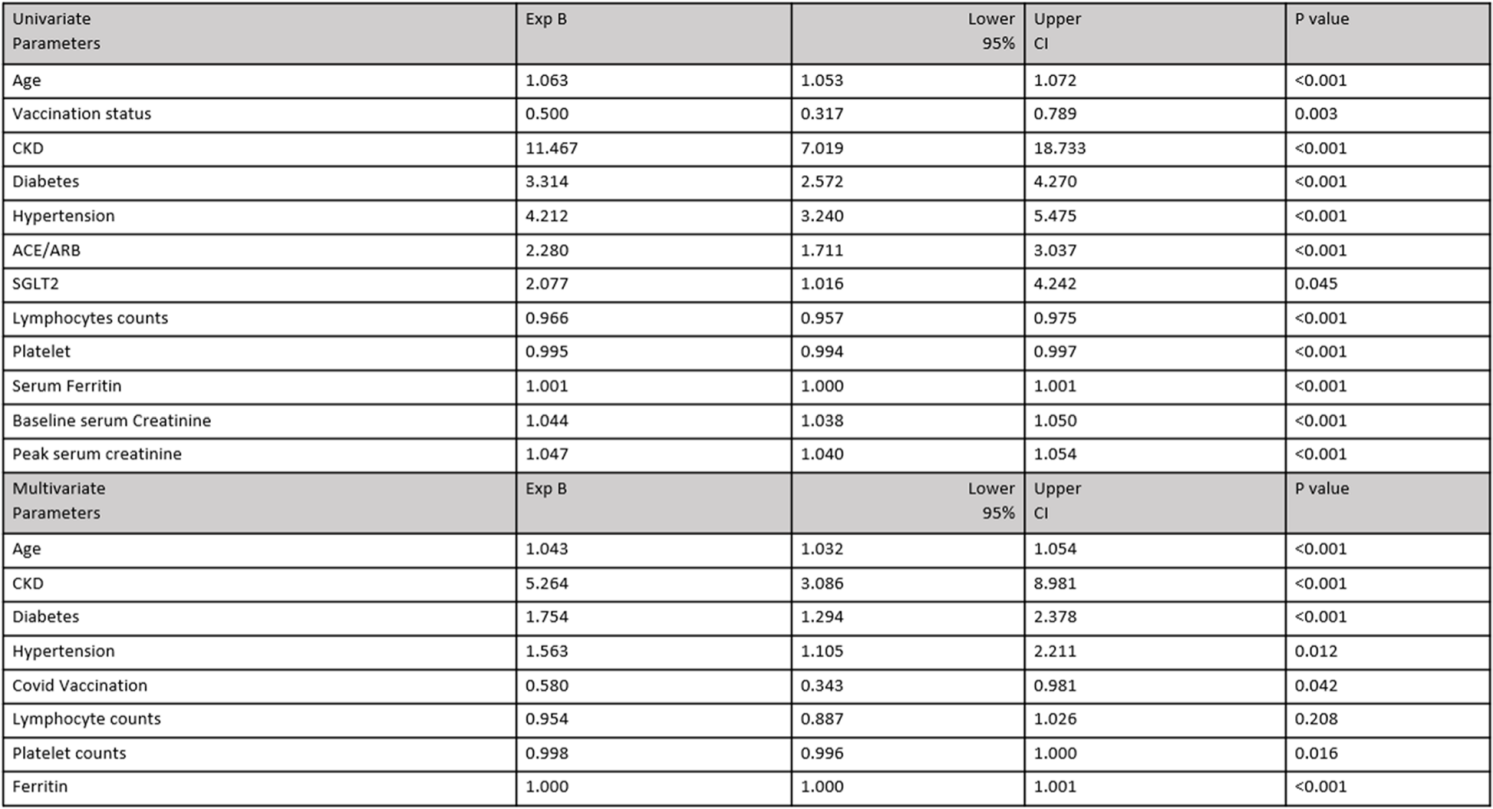
Summarizes the univariate and multivariate logistic regression for the predictors of AKI in hospitalized COVID-19 patients

## Discussion

Virus infection can lead to a wide range of kidney diseases through the heterogeneous mechanisms, and virus-associated AKI is well-known phenomenon [10]. Major epidemics such as Severe acute respiratory syndrome (SARS) and Middle East Respiratory Syndrome coronavirus (MERS-COV) mainly affect the respiratory system. Interestingly, the aforementioned infections commonly afflict the kidney and have been associated with long-term adverse renal outcomes. A retrospective study by Kwok et al. demonstrated that in 2003, the incidence rate of AKI was 6.7% in SARS infection and was associated with a higher mortality rate of 91.7% [11]. In addition, during the outbreak of Middle East Respiratory Syndrome coronavirus (MERS-COV), up to 58% of the patients admitted to the intensive care unit needed renal replacement therapy for AKI in Saudi Arabia [12].

The incidence of AKI due to COVID-19 has been reported to vary worldwide, ranging from 5%- 56% [13, 14]. Meta-analysis revealed that the observed difference might be due to geographical variation and the proportion of severely ill patients reported in each study. Western countries reported a higher incidence of AKI in COVID-19 infection than Asian countries. An observation study in New York reported 46% of AKI among the hospitalized COVID-19 infection, with the proportion of white, black, and Asian being 24%, 29%, and 4%, respectively [15]. Jewell et al. revealed that 39% of hospitalized COVID-19 developed AKI, with blacks having the highest proportion rate (46%) followed by whites (36%) and Asians (32%) in London [16]. In contrast, another retrospective study in the United Kingdom reported a lower rate of AKI (20.3%) among hospitalized COVID-19 patients, but the majority of the admissions were of white ethnicity, with 70.3% white, 9.1% black and 11% Asian, that’s markedly different from the Jewell et al. cohort [17]. Thus, the incidence rate of AKI in Western countries seemed to depend on the study's proportion of black ethnicity. Of note, Asian ethnicity tends to have a lower incidence rate of AKI in Western countries. This is further supported by a subgroup analysis of a systemic review that reported a two-fold rise in the incidence of AKI in non-Asian populations, especially those of African ancestry, significantly more than the Asian population [18]. Our Asian cohort mainly consists of Malay, Chinese, and Indian ethnicities without black or African ancestry and hence explaining the lower incidence of AKI. In our study, we noted 323 out of 1529 patients (21.1%) developed AKI, which is lower than the reported incidence rate from Western countries.

In our study, the classification of AKI severity is based on KDIGO criteria 2012. We noted that most hospitalized patients who developed AKI predominantly fell into stage 1 (77%), which is in keeping with findings from most other studies indicating stage 1 AKI was the commonest occurrence [14, 19–21]. Severe AKI (stage 2 or 3) was more prevailing in critically ill patients who needed intensive care [22, 23] and it was associated with higher mortality. Indeed, the risk of mortality increases with the severity of AKI in COVID-19 patients, as demonstrated by ISARIC WHO CCP-UK Study which showed that AKI stage 1 has an odd ratio (OR) of 1.58 (95% CI 1.49- 1.67) on 28-day mortality, and the OR rose further to 3.50 (95% CI 3.14–3.91) for AKI stage 3 [20]. In our study, a 34.7% mortality rate was observed in those who had AKI across all spectrums of stages. Our findings are consistent with the reported mortality rate in the study by Morieri M et al., which demonstrated a mortality rate of 39.3% in AKI patients, confirming that AKI was an independent predictor for mortality after adjusting for cofounders [24]. The noted poor outcome in our analysis may be due to the difficulty in caring for an overwhelming number of COVID-19 patients in a single center with limited resources such as beds, staffing, and equipment for renal replacement therapy during the peak of the COVID-19 pandemic.

Urinary abnormalities, including proteinuria, hematuria, and leukocyturia, are seen in hospitalized COVID-19 patients. In our study, not all the patients had urinalysis; however, among those who had urine examination, approximately 56% of the non-AKI patients had either proteinuria or microscopic hematuria, leukocyturia, whereas a higher (92%) of AKI COVID-19 patients had mentioned urinary disorders, thus concurring with Chan et al. who reported a greater proportion of urinalysis abnormalities in patients with AKI, as the author reported 80% had either proteinuria or hematuria, and 60% had leukocyturia [15]. Thus, this highlights the importance of urinalysis for hospitalized COVID-19 patients, as the presence of proteinuria/microscopic hematuria has been reported to be an independent risk factor for AKI, morbidity, and mortality [13].

In light of AKI and urinary abnormalities on adverse patients’ outcome, the importance of understanding the mechanism of AKI in COVID-19 has been prioritized and intensively studied, with the intention of devising a proper preventive measure and treatment strategies. Kidney disease among COVID-19 patients may manifest with different clinical phenotypes. The consensus report of the 25th Acute Disease Quality Initiative (ADQI) work group postulated different mechanisms through which COVID-19 contributes to AKI [25]. The direct effect of viruses resulting in collapsing glomerulopathy in renal has been well-reported in case studies [26–29]. Multisystem inflammatory syndrome (MIS-A) related to COVID-19 infection has been described [30], postulating hyperinflammation, uncontrolled complement activation, and endothelial dysfunction having direct insult to the kidney structures. A review article by Sharma P et al. delineates the pathological changes of COVID-AKI due to various mechanisms with acute tubular injury, collapsing glomerulopathy, and thrombotic microangiopathy frequently seen in both living and autopsied renal tissues. Other less common findings noted were podocytopathies, lupus nephritis, anti-glomerular basement membrane disease and anti-neutrophil cytoplasmic antibody vasculitis [31]. Most importantly, indirect injury to the renal system, e.g., hypovolemia, sepsis-associated AKI, acute tubular necrosis, acute interstitial nephritis due to nephrotoxic medications, and increased positive end-expiratory pressure ventilator induce renal angiotensin activation aldosterone system, is thought to be the major culprits.

Our cohort revealed that severe AKI requiring dialysis during hospitalization was low (4.6%) among the AKI patients (15 out of 323 patients); however, more than one-third of the patients had no renal recovery during the follow-up review. A study in New York City showed that, overall, 19% of COVID-19 patients needed RRT, and a third of patients had no renal recovery from COVID-19 [15]. In contrast, a prospective observational study of 4613 patients with COVID-19 AKI in India reported a high RRT rate of 40.5% with a high non-recovery rate of 72% upon discharge till three months follow-up, and 49% of the patients eventually progressed to chronic kidney disease [32]. The disparity in dialysis requirement and the rate of renal recovery in various centers could be attributed to several factors. The most likely cause is the different thresholds of initiating RRT in different centers. Centers with lower thresholds for dialysis may potentially have worse renal outcomes. This is supported by a retrospective study illustrating the rate of renal recovery in severe AKI needing dialysis had a renal recovery rate of 61% on days 1–4 and 8% in 31–90 days [33]; Other factors may be related to the variation of the duration of the post-hospitalization follow-up and definition of renal recovery thus leading to the disparity in the reported results. This emphasizes the importance of understanding the pathophysiology and risk factors of COVID-related AKI and the clinical progress post-COVID-19 recovery. The association of non-recovery AKI and evolution to chronic kidney disease is well-known in other diseases [34, 35]; however, whether a similar theory applies to COVID-19 AKI is uncertain as of now. In the event of a high rate (≈30%) of non-renal recovery COVID-19 AKI, we foresee the clinical challenges of dealing with an increasing chronic kidney disease population. Thus, serial monitoring of the renal function in hospitalized COVID-19 patients is crucial, and those with AKI will require a longer period of review to determine renal recovery. This will allow the implementation of preemptive measures involving the prevention and retardation of chronic kidney disease.

With the aforementioned high proportion of non-recovery AKI in COVID-19 underscores the importance of early prediction of the clinical phenotype by analyzing factors associated with AKI hospitalized COVID-19. In this study, we dissected the risk factors based on demographic and clinical parameters and found that age and pre-existing CKD were statistically significant in predictors of AKI in hospitalized COVID. These findings concur with other reports indicating age as an independent predictor for AKI [14, 36, 37]. Furthermore, age has also been reported as a risk factor for death in COVID-19 patients [19, 38], suggesting that elderly population is at a higher risk of morbidity and mortality, warranting special attention upon admission. In addition, similar to our study cohort, a retrospective study in London demonstrated a three-fold risk of AKI for patients who developed COVID-19 AKI with pre-existing CKD [16]. Diabetes and hypertension is highly prevalent worldwide [39, 40]. Patients who contracted COVID-19 with underlying comorbidities were associated with poorer outcomes [41, 42]. In our cohort, patients with either diabetes or hypertension were independent predictors for AKI, and our findings were supported by other studies [43, 44]. In addition, ferritin, an acute phase reactant, tends to be elevated in inflammatory conditions, including infections. Reports have shown that a raised ferritin level can predict AKI among COVID-19 patients, and this is consistent with our cohort [45, 46]. The ROCs of serum ferritin in predicting AKI are shown in Fig. 3 with an AUC of 0.711, representing a moderate ability to discriminate prediction for the development of AKI. An optimal cutoff point of 500 ug/l in our analysis allows a performance characteristic of a positive predictive value [PPV] of 34% and a negative predictive value [NPV] of 87%. Our study did not track the ferritin trend during hospitalization, unlike Jonathan Feld et al., who demonstrated ferritin levels at different time frames may be useful as a predictor for the need for renal replacement therapy (RRT) among hospitalized COVID-19 patients. His study reported the presentation of ferritin with a threshold above 569 ng/ml (sensitivity of 0.84 and specificity of 0.46) and maximum ferritin of more than 2365 ng/ml (sensitivity of 0.62 and specificity of 0.74) are useful as a predictors for severe AKI requiring RRT [47]. Thrombocytopenia is a well-known clinical sequela in viral infection [48]. However, the reported incidence rates were less in COVID-19 patients ranged from 5–41.7% [49, 50]. The seemingly lower number may be due to the distinct clinical phenotype of COVID-19 to other types of viral infection as it is associated with hypercoagulable state. There are various possible mechanisms of COVID-19-associated thrombocytopenia, including platelet activation, platelet clearance, platelet autoantibody formation, splenic sequestration, and marrow suppression [51]. Our study showed that the incidence rate of thrombocytopenia was two times higher in the AKI group (*n* = 73; 22.6%) than AKI-naïve group (*n* = 126; 10.4%); *p* < 0.001 even though the median platelet counts were within the normal range for both groups. The apparent normal platelet count may be explained by a corresponding increase in platelet production despite an increase platelet consumption and hyperreactivity. This finding concurs with Taha et al., that reported increased platelet activation in COVID-19 patients as measured by a higher mean platelet volume despite a normal platelet count. In his study, there was a high incidence of AKI in critically ill patients with unique platelet features, that including larger, more granular platelets and a higher mean platelet volume [52]. We found that platelet counts were an independent predictor for AKI in hospitalized COVID-19 patients.

Most experts think that vaccine is the most effective way of curbing the global spread of viral disease that including Covid-19 infection. Intensive research was undertaken in the early phase of the pandemic, and various types of COVID-19 vaccines were being developed and listed for emergency use by WHO, which comprised mRNA vaccine (Pfizer BioNTect), whole virus vaccine (Sinovac) and non-replicating viral vector (Oxford-AstraZeneca). Our study revealed that Pfizer vaccination had a significantly positive renal outcome, with the majority of the vaccinated patient (90.6%) not developing AKI. Patients who received either AstraZeneca (18.5%) or Sinovac (21.4%) vaccines also had a lower risk of developing in hospitalized COVID-AKI, albeit it was not statistically significant in our study. We believe that the immunogenicity of various types of COVID vaccines may confer a different protective effect from acute kidney injury; however, this will require further analysis.

Despite the perceived positive effect of the COVID-19 vaccine on renal outcome in our cohort, there is a potential pitfall with the review article by Yebei Li which highlighted 52 cases of Acute Kidney Disease (AKD) that encompassing podocytopathy, IgA nephropathy, vasculitis, tubular interstitial nephritis and thrombotic microangiopathy following the Covid vaccination [53] The causal relationship between COVID-19 vaccination and AKI is uncertain for now, however considering the low rate of vaccine-induce AKI, coupled with the significant risk reduction of adverse patient outcomes related to COVID-19 infection, it would be sensible to implement vaccination in a population but with the understanding of the potential side effect of the vaccines.

There are a few limitations in our study. Firstly, it is a retrospective study, and not all the patients have the baseline serum creatinine. Hence, we used the on-arrival serum creatinine at the emergency department for those who lacking baseline serum creatinine. In our study, the timing of post-discharge serum creatinine varied substantially, ranging from two weeks up to three months after being discharged from the ward. Therefore, we may prematurely label some patients as having no renal recovery if the latest post-discharge serum creatinine was obtained before three months after the AKI episode. Furthermore, the timing of AKI development during hospitalization was not consistently recorded, even though the author believe it is important to look for the temporal relationship between the onset of AKI and the severity of COVID-19 infection. The diagnoses of pneumonia and acute respiratory distress syndrome (ARDS) were based on the treating physician or anesthetist assessment rather than interpretation by an expert such as a radiologist or pulmonologist. Despite these limitations, a large sample size of more than a thousand patients in this study provides robust data reflecting the Malaysian population.

In conclusion, the incidence of AKI is not rare among hospitalized COVID-19 patients and is associated with prolonged hospitalization, poorer renal outcomes, and higher mortality. Our study suggests that COVID-19 vaccination reduces the incidence rate of AKI, but future studies are warranted to look for the long-term AKI outcome in the COVID-19 vaccinated patients. Given the adverse impact of AKI on COVID-19 patients, earlier detection of predictors, including the demographic factors and clinical parameters, is essential to improve the patient renal and survival outcomes.

## Data Availability

The datasets used and/or analyzed during the current study are available from the corresponding author upon reasonable request.

## References

[1] Abate SM, Checkol YA, Mantefardo B (2021). Global prevalence and determinants of mortality among patients with COVID-19: a systematic review and meta-analysis. Ann Med Surg (Lond).

[2] Ng WH (2021). Comorbidities in SARS-CoV-2 patients: a systematic review and meta-analysis. MBio.

[3] McGrowder DA (2021). Abnormal liver biochemistry tests and acute liver injury in COVID-19 Patients: current evidence and potential pathogenesis. Diseases.

[4] Li S (2022). Clinical characterization and possible pathological mechanism of acute myocardial injury in COVID-19. Front Cardiovasc Med.

[5] Sarubbo F (2022). Neurological consequences of COVID-19 and brain related pathogenic mechanisms: a new challenge for neuroscience. Brain Behav Immun Health.

[6] Agbuduwe C, Basu S (2020). Haematological manifestations of COVID-19: From cytopenia to coagulopathy. Eur J Haematol.

[7] Jevnikar K (2021). An update on COVID-19 related ophthalmic manifestations. Ocul Immunol Inflamm.

[8] Staff R (2020). Malaysia confirms first cases of coronavirus infection.

[9] Portal, D.o.S.M.O. Current Population Estimates, Malaysia, 2022. 2022 25 July 2022]; Available from: https://www.dosm.gov.my/v1/index.php?r=column/cthemeByCat&cat=155&bul_id=dTZXanV6UUdyUEQ0SHNWOVhpSXNMUT09&menu_id=L0pheU43NWJwRWVSZklWdzQ4TlhUUT09.

[10] Masset C (2022). Virus-associated nephropathies: a narrative review. Int J Mol Sci.

[11] Chu KH (2005). Acute renal impairment in coronavirus-associated severe acute respiratory syndrome. Kidney Int.

[12] Arabi YM (2014). Clinical course and outcomes of critically ill patients with Middle East respiratory syndrome coronavirus infection. Ann Intern Med.

[13] Cheng Y (2020). Kidney disease is associated with in-hospital death of patients with COVID-19. Kidney Int.

[14] Fisher M (2020). AKI in hospitalized patients with and without COVID-19: a comparison study. J Am Soc Nephrol.

[15] Chan L (2021). AKI in hospitalized patients with COVID-19. J Am Soc Nephrol.

[16] Jewell PD (2021). Correction to: COVID-19-related acute kidney injury; incidence, risk factors and outcomes in a large UK cohort. BMC Nephrol.

[17] Hamilton P (2020). Characteristics and outcomes of hospitalised patients with acute kidney injury and COVID-19. PLoS ONE.

[18] Xu Z (2021). Systematic review and subgroup analysis of the incidence of acute kidney injury (AKI) in patients with COVID-19. BMC Nephrol.

[19] Gameiro J (2021). Acute kidney injury in hospitalized patients with COVID-19: a Portuguese cohort. Nefrologia (Engl Ed).

[20] Sullivan MK (2022). Acute kidney injury in patients hospitalized with COVID-19 from the ISARIC WHO CCP-UK Study: a prospective, multicentre cohort study. Nephrol Dial Transplant.

[21] Hirsch JS (2020). Acute kidney injury in patients hospitalized with COVID-19. Kidney Int.

[22] Lumlertgul N (2021). Acute kidney injury prevalence, progression and long-term outcomes in critically ill patients with COVID-19: a cohort study. Ann Intensive Care.

[23] Hsu CM (2022). Kidney recovery and death in critically ill patients with COVID-19-associated acute kidney injury treated with dialysis: The STOP-COVID Cohort Study. Am J Kidney Dis.

[24] Morieri ML (2022). In hospital risk factors for acute kidney injury and its burden in patients with Sars-Cov-2 infection: a longitudinal multinational study. Sci Rep.

[25] Nadim MK (2020). COVID-19-associated acute kidney injury: consensus report of the 25th Acute Disease Quality Initiative (ADQI) Workgroup. Nat Rev Nephrol.

[26] Su H (2020). Renal histopathological analysis of 26 postmortem findings of patients with COVID- 19 in China. Kidney Int.

[27] Hassler L (2021). Evidence for and against direct kidney infection by SARS-CoV-2 in patients with COVID-19. Clin J Am Soc Nephrol.

[28] Wu H (2020). AKI and collapsing glomerulopathy associated with COVID-19 and APOL 1 high- risk genotype. J Am Soc Nephrol.

[29] Kissling S (2020). Collapsing glomerulopathy in a COVID-19 patient. Kidney Int.

[30] Patel P (2021). Clinical characteristics of multisystem inflammatory syndrome in adults: a systematic review. JAMA Netw Open.

[31] Sharma P (2021). Pathology of COVID-19-associated acute kidney injury. Clin Kidney J.

[32] Bansode J (2022). Acute kidney injury in COVID-19: clinical profile and outcome. Indian J Nephrol.

[33] Siew ED (2020). Timing of recovery from moderate to severe AKI and the risk for future loss of kidney function. Am J Kidney Dis.

[34] Chawla LS (2011). The severity of acute kidney injury predicts progression to chronic kidney disease. Kidney Int.

[35] Macedo E, Zanetta DM, Abdulkader RC (2012). Long-term follow-up of patients after acute kidney injury: patterns of renal functional recovery. PLoS ONE.

[36] Costa RLD (2021). Acute kidney injury in patients with Covid-19 in a Brazilian ICU: incidence, predictors and in-hospital mortality. J Bras Nefrol.

[37] Farooqui MA (2021). Incidence and outcome of acute kidney injury in patients hospitalized with coronavirus disease-19 at a Tertiary Care Medical Center in Saudi Arabia. Cureus.

[38] Goh BL (2022). COVID-19 death and kidney disease in a multiracial Asian country. Nephrology (Carlton).

[39] Organization, W.H. Diabetes. 2022. [16 September 2022]. Available from: https://www.who.int/news-room/fact-sheets/detail/diabetes.

[40] Organization, W. H. Hypertension .2021. [25 August 2021]; Available from: https://www.who.int/news-room/fact-sheets/detail/hypertension.

[41] Khedr EM, et al. Impact of comorbidities on COVID-19 outcome. 2020. medRxiv.

[42] Bae S (2021). Impact of cardiovascular disease and risk factors on fatal outcomes in patients with COVID-19 according to age: a systematic review and meta-analysis. Heart.

[43] Cai X (2021). Risk factors for acute kidney injury in adult patients with COVID-19: a systematic review and meta-analysis. Front Med (Lausanne).

[44] Oweis AO (2022). Acute kidney injury among hospital-admitted COVID-19 patients: a study from Jordan. Int J Gen Med.

[45] Marques F (2021). Acute kidney disease and mortality in acute kidney injury patients with COVID-19. J Clin Med.

[46] Fabrizi F (2022). Acute kidney injury in Non-Intensive Care Unit (ICU) hospitalizations for coronavirus disease (COVID-19). Pathogens.

[47] Feld J (2020). Ferritin levels in patients with COVID-19: a poor predictor of mortality and hemophagocytic lymphohistiocytosis. Int J Lab Hematol.

[48] Klinger MH, Jelkmann W (2002). Role of blood platelets in infection and inflammation. J Interferon Cytokine Res.

[49] Tang N (2020). Abnormal coagulation parameters are associated with poor prognosis in patients with novel coronavirus pneumonia. J Thromb Haemost.

[50] Mei H, Hu Y (2020). Characteristics, causes, diagnosis and treatment of coagulation dysfunction in patients with COVID-19. Zhonghua Xue Ye Xue Za Zhi.

[51] Wool GD, Miller JL (2021). The impact of COVID-19 disease on platelets and coagulation. Pathobiology.

[52] Taha M (2021). Platelets and renal failure in the SARS-CoV-2 syndrome. Platelets.

[53] Li Y, Rao M, Xu G (2022). New-onset acute kidney disease post COVID-19 vaccination. Vaccines (Basel).

